# History of a Case of Partial Reconstruction of the Face

**Published:** 1865-04

**Authors:** 


History of a Case of Partial Reconstruction of the Face. By Gurdon Buck, M.D., Surgeon to the New York Hospital, St. Luke's Hospital, &c. &c. Reprinted by permission, from the Transactions of the New York State Medical Society.

This is a pamphlet of 16 pages, to which are added six or seven photographic plates. It gives an account of one of the most successful efforts to repair an extensive injury of the face, that is to be found in our surgical literature. We would copy it entire, but much of its value and interest would be lost without the accompanying plates.

